# Towards person-centered care in global mental health: implications for meta-analyses and clinical trials

**DOI:** 10.1017/S2045796025000071

**Published:** 2025-02-21

**Authors:** Davide Papola, Vikram Patel

**Affiliations:** 1 WHO Collaborating Centre for Research and Training in Mental Health and Service Evaluation, Department of Neuroscience, Biomedicine and Movement Science, Section of Psychiatry, University of Verona, Verona, Italy; 2 Department of Global Health and Social Medicine, Harvard Medical School, Boston, MA, USA

**Keywords:** Evidence-Based Psychiatry, health outcomes, mental health, quality of care

## Introduction

Mental health accounts for a significant proportion of the global burden of disease, yet it remains chronically underfunded and underserved, especially in resource-poor settings (Patel *et al.*, 2018). In response, the field of global mental health has emerged to champion scholarship, education and advocacy to address mental health equity globally and within countries (Patel and Prince, 2010). While the global mental health agenda covers a range of multisectoral actions from structural interventions to community interventions (Papola *et al.*, 2024a), a major focus has been on clinical research to improve access and outcomes to psychosocial interventions in routine care settings. A substantial body of evidence has been generated through randomized controlled trials (RCTs), particularly for mood, anxiety and trauma-related mental health conditions, which are then typically quantitatively synthesized through standard pairwise meta-analyses pooling aggregate-level study data (Singla *et al.*, 2017).

Such meta-analyses have influenced clinical practice and policymaking in several fields. One example is the utilization of antenatal corticosteroids to accelerate fetal lung maturation in women at risk of preterm birth, a finding so important that the Cochrane Collaboration incorporated the forest plot of the main analysis into its own logo (Reynolds and Tansey, 2005). Global mental health is no exception, having also relied on meta-analyses to synthesize clinical evidence from both randomized and observational studies (Papola *et al.*, 2024b, 2020; Purgato *et al.*, 2018a). On the other hand, the volume of published conventional meta-analyses has exploded in recent decades, even outpacing the production of primary research, raising concerns about their quality and necessity (Ioannidis, 2016a). This trend is driven by several factors, including the relatively low resource requirements, both in terms of time and funding, for conducting meta-analyses and the fact that they tend to be highly cited (Rawat and Meena, 2014). Although stricter standards for transparency in reporting have been implemented (Page *et al.*, 2021), the ‘mass production of redundant, misleading, and conflicted meta-analyses’ has generated criticism and fuelled skepticism about their overuse, thus diluting credibility (Ioannidis, 2016b). Furthermore, in an era where clinical practice demands personalization, the ability of standard pairwise meta-analytic methods to generate breakthrough insights is waning.

As articulated by the Lancet Commission on Global Mental Health and Sustainable Development, person-centered care is about tailoring care to an individual’s specific characteristics, needs, circumstances, preferences, and outcomes while emphasizing the dimensionality of psychological distress, the interplay between social and individual determinants of mental health, and the contextual nature of diagnostic and intervention paradigms (Patel *et al.*, 2018). In this article, we explore how five key methodological considerations should be incorporated into the design of future meta-analytic analyses and RCTs.

## From group-level analyses to precision analyses of individual outcomes

The ultimate goal of precision medicine is to examine ‘what works, for whom, and under what conditions’ (Kiesler, 1966; Paul, 1967). This requires RCTs to be sufficiently powered to address sub-group effects of experimental interventions, which increases sample sizes exponentially to detect interactions with baseline moderators (Brookes *et al.*, 2004). Indeed, it is difficult to find a single example from the vast number of RCTs conducted to test the efficacy of psychotherapy for depression or anxiety that is adequately powered to examine a moderator (Cuijpers *et al.*, 2022a, 2022b). In addition, RCTs are rigid by design, typically assessing the effect of an intervention and providing information on mean differences between comparison groups so that the conclusions of the study apply more to an ‘average patient’ who met the criteria for inclusion in the study than to the heterogeneous range of patients encountered in routine clinical practice (Feinstein and Horwitz, 1997). Such aggregate analyses imply that the most effective intervention at the group level may not be the most effective for a particular patient (a problem that is compounded in traditional pairwise meta-analyses of aggregate data). A major methodological advance from the traditional pairwise meta-analytic technique, which promises to move the field from traditional one-size-fits-all approaches towards person-centered care, is represented by individual participant data meta-analysis (IPDMA) (Veroniki *et al.*, 2023). The advent of sharing of individual patient-level data has ushered the opportunity to harmonize multiple RCT datasets addressing the same research question to create a ‘megatrial’ of samples that are exponentially larger than any individual trial. For a patient of interest (i.e., with certain baseline characteristics such as gender or severity of psychopathology), similar participants from different but comparable clinical trials are pooled so that outcomes for that subpopulation can be analysed to estimate a personalized risk-benefit ratio and the impact of participant-level prognostic factors (baseline characteristics that predict outcome regardless of intervention) and effect modifiers (covariates that predict differential response to treatments) on intervention outcomes (Purgato *et al.*, 2018b; Karyotaki *et al.*, 2022). This information can be further enriched and refined by machine learning predictive models, such as, for e.g., imputation techniques that use chained random forest algorithms for the handling of missing data (Mayer, 2024). Machine learning facilitates the identification of complex, non-linear interactions and patterns within heterogeneous datasets and is adept at identifying predictors and interactions that provide robust risk stratification for the development of personalized treatment strategies (Chakraborty *et al.*, 2024). These approaches lie at the heart of the personalized medicine initiative, which aims to tailor interventions not only to a patient’s clinical picture but also to their other characteristics (Johnson *et al.*, 2021). However, a major constraint to fully realize the potential of these techniques is the lack of consistency with which baseline co-variates are measured and the standardization of instruments to measure these co-variates. Such constraints relating to the assessment of mental health outcomes have recently been addressed by major donors such as the Wellcome Trust, requiring all trials funded by them to use a basic standardized set of outcome measures. We recommend a similar approach for baseline characteristics, identifying a minimum set of covariates based on both emerging evidence from IPDMAs and expert consensus to ensure that the next generation of RCTs can fully inform future IPDMAs.

## From overall intervention effects to effectiveness at the level of components

Most psychosocial interventions are eclectic compilations of multiple, distinct, possibly interacting, array of practices spanning behavioural, interpersonal, cognitive and emotional domains, as well as psychoeducation and social work elements. These are, in turn, layered upon a set of ‘foundational’ components, such as the counselor’s attitude, that are common to all psychosocial interventions. While conventional meta-analyses of RCTs show that psychosocial interventions are effective in preventing and treating mental health problems (Van Ginneken *et al.*, 2021), they are unable to unpack which components are driving the response. Thus, the ‘package’ level of evaluation is too broad and imprecise to answer more targeted, specific questions about the mechanisms of change or personalization. Further, nearly all trials evaluating psychosocial interventions compare them against inactive comparisons, and there is a lack of studies comparing interventions head-to-head or dismantling studies that examine the efficacy of individual components. As a result, the current literature on the efficacy of psychosocial interventions throws up inconsistent findings, with dozens – if not hundreds – of varieties of psychosocial interventions with overlapping components tested in trials comparing a single intervention to a no-intervention comparison for a single disorder. Unpacking the effectiveness of specific components of psychosocial interventions is not only an essential step in understanding how such interventions work but also holds the promise of simplifying complex packages by stripping off those components which are ineffective. Component network meta-analyses (cNMAs) compare the components of different therapies by exploiting the randomized structure of the evidence – i.e., the intervention effects are estimated separately in each trial, and then the trial-specific estimates are pooled across the network (Rücker *et al.*, 2020). Moreover, these methods can be arranged in multiple comparisons, grafting IPD and/or component analyses onto a ‘network’ structure, allowing the simultaneous comparison of multiple interventions, even when these are not directly compared. The cNMA statistical approach ‘dismantles’ each composite intervention, first by modelling the effects of specific components and then by combining them in two ways: (a) additively, which assumes that the components do not interact and that the overall intervention effect is additive; (b) interactively, which examines combinations of components. The result is a network of comparisons and a hierarchy that includes all intervention components, expressed as a measure of ‘incremental risk’, which indicates the added benefit (or disadvantage) of adding an active ingredient to an intervention (Tsokani *et al.*, 2022). A key requirement for approximating causal inference is that the dismantling of interventions is exhaustive, covering all components in the included interventions and that each component is coherent in terms of its intended target and the activities it contains. Component taxonomies that could be used for component analyses have been proposed in mental health (Abraham and Michie, 2008; Chorpita and Daleiden, 2009; Singla *et al.*, 2017; Pedersen *et al.*, 2020). An updated taxonomy for psychosocial interventions specifically conceived and developed for quantitative measurement is in preparation (Papola *et al.*, 2023) and we recommend that all future trials of complex multi-component psychosocial interventions use such a taxonomy to describe the constituent components and measure their delivery. In addition, we recommend trials that evaluate the effectiveness of specific components, similar to what has been done with the Healthy Activity Program (whose primary component is behavioural activation) (Patel *et al.*, 2017), including those in comparison to other components.

## From categorical or diagnostic assessments to dimensional assessment of suffering

Mental disorders are not natural ontological entities separated from each other by clear boundaries but abstract constructs that share psychopathological features that cut across them. Despite this fact, mental health research and practice have adopted a reductionist approach to nosology, categorizing syndromes of psychopathology based on observed differences in clinical phenotype rather than aetiological distinctiveness. Disappointingly, fifty years of research using this diagnostic system has failed to identify the aetiology of any diagnostic category, both because of the immense heterogeneity within diagnostic categories and the blurred boundaries between them. For example, depression and anxiety share similar epidemiological profiles, often co-occur and respond to similar treatments, and can be measured by scales that capture both symptomatologies (Derogatis *et al.*, 1974; Zigmond and Snaith, 1983), even though they are categorized as separate entities in diagnostic manuals (Kalin, 2020). While the deployment of an algorithmic diagnostic approach has certainly improved the reliability of psychiatric diagnosis and aligned mental health practice with other medical disciplines, it has done so at the expense of embracing the reality of the dimensional and overlapping nature of psychopathology, ultimately failing to capture differences in individual experience (Ritunnano *et al.*, 2023; Reininghaus *et al.*, 2024). One-size-fits-all approaches have failed to deliver better outcomes because care should be based on a person’s needs, not their diagnosis (Insel, 2022). We recommend that meta-analyses recode diagnostic groups using broad dimensions of psychopathology, such as those described in the HiTOP framework (Conway and Forbes, 2022), and which take into account the spectrum nature of suffering within each of these dimensions ranging from mild, often self-limiting suffering to chronic and refractory disorders (Mcgorry *et al.*, 2014), and that future trials also adopt transdiagnostic and dimensional frameworks, to define eligibility for participation. Relatedly, clinical outcomes should be assessed using transdiagnostic measures, such as the Kessler scales (Kessler *et al.*, 2002).

## From solely evaluating clinical outcomes to embracing person-centered outcomes

The vast majority of RCTs and, consequently, meta-analyses, focus on clinical outcomes. Apart from the limitation of such outcomes as noted above, there is a growing body of evidence that demonstrates that an alternative approach is to adopt a person-centered perspective that is deeply intertwined with individual, social and economic circumstances, moving away from narrow biomedical categorizations (Patel *et al.*, 2023). Person-centered outcomes in mental health prioritize individual experiences, preferences and goals. These outcomes include recovery-oriented outcomes such as quality of life, quantity and quality of social connections, subjective well-being, self-efficacy, hope, prosocial behaviour and daily psychosocial functioning (Papola *et al.*, 2024b). While such outcomes are often measured in RCTs, they are rarely considered primary outcomes either in individual trials or for meta-analyses. Examples exist of how person-centered outcomes can be framed as co-primary outcomes, as in the case of evaluating a problem-solving psychological intervention for adolescents with emotional disorders on both clinical outcomes and self-rated problem severity (Michelson *et al.*, 2020; Malik *et al.*, 2021). We recommend that meta-analyses consider such outcomes, even if categorized as secondary, alongside traditional clinical outcomes and that future trials add person-centered outcomes as co-primary outcomes.

## From income-based setting classification to more nuanced contextual characterization

Global health has historically focused on improving access to treatment in settings where the treatment gap is extremely high. To delineate the contexts of interest, the World Bank’s criteria for classifying countries based on their gross domestic product (GDP) have been widely adopted, and much global health research has focused on the so-called ‘low- and middle-income countries’ (LMICs), which may also be referred to as ‘developing countries’ or ‘countries of the global south’, based on the assumption that these countries are resource-poor. However, the dichotomization of high-income countries (HICs) versus LMICs implies homogeneity within each grouping and assumes that a country’s economic productivity is the primary driver of health. However, most of the world’s population lives in LMICs, each of which is at different stages of economic and social progress, with equally large variations within countries, for example, depending on where the population lives. These differences are predicated on variations in a range of human development indicators that go well beyond a crude categorization of GDP and which have a great bearing on the prevalence and response to mental health problems. While HIC tends to be more homogenous, there are also significant inequities within these countries, both geographical and related to sub-groups in the population. Thus, we propose the use of the term ‘low-resource settings’ which acknowledges that disparities in mental health exist in all countries and reaffirms that ‘all countries can be considered developing when it comes to mental health’ (Patel et al., 2018; The Lancet Global Health, 2020). Such resource-based approaches can help galvanize the synthesis of evidence and its generalization and uptake across contexts that are similar, for example, through meta-analyses that focus on ‘low-resourced’ settings. Such an approach is also aligned with the concept of ‘glocal’, as exemplified by a new generation of studies in HIC which are implementing psychosocial interventions and delivery approaches developed in LMICs (Anand and Pai, 2023). A resource-based approach for describing context should focus on the overall availability of and access to public goods (for example, education and healthcare) and socio-demographic profiles, for example, using the framework designed by van Zyl et al., which operationalized nine themes related to the resource-based approach (Van Zyl *et al.*, 2021) or the United Nation’s Human Development Index which classifies countries and sub-national contexts on a range of indicators measuring standards of living, life expectancy, education, access to technology, food security and environmental quality (United Nations Development Programme, 2023). We recommend that meta-analyses consider inclusion criteria which can capture such nuanced contextual diversity and that all future trials incorporate such categorization in the description of their samples. A resource-based approach would recognize that evidence and actions in mental health must be tailored to specific contexts, regardless of national income status, to reduce inequalities.

## Conclusion

Epidemiological and clinical research has convincingly demonstrated that mental disorders are profoundly influenced by the interaction of a range of individual and contextual characteristics. Furthermore, these disorders should not be viewed as discrete categorical entities with distinct aetiologies, but rather as consisting of often overlapping dimensions along which each individual may have different experiences and move dynamically. These findings lie at the heart of observations of wide variation in patient experience within diagnostic categories, and are arguably the main reason why evidence from trials evaluating the effectiveness of complex psychosocial interventions for specific diagnostic groups has not been consistently replicated, why effective interventions do not help all patients with a given condition, and why meta-analyses often produce conflicting results. The future of evidence-based global mental health research, both individual RCTs and meta-analyses, must incorporate methodological innovations to address these challenges. We have identified five such innovations: measuring a range of patient characteristics that can be used in IPDMAs to assess which types of patients respond to specific interventions; unpacking the ‘black box’ of multicomponent psychosocial interventions by characterizing the specific components of the intervention and assessing the effectiveness of single-component interventions; characterizing the mental health problem using transdiagnostic and dimensional frameworks; including person-centered outcomes along with clinical outcomes; and characterizing the context of a study using more nuanced resource-based criteria rather than GDP. By adopting a component and individual patient data approach, it is possible to examine which components are most effective and for whom, how individual social determinants of mental health, level of available resources, provider and delivery characteristics moderate the outcome, and how beneficial effects of interventions are mediated. Then, by focusing on a trans-diagnostic perspective, adopting a dimensional perspective and incorporating person-centered outcome measures, it is possible to calibrate meta-analytic findings with the diversity of clinical presentations in community and primary care settings. In this way, both RCTs and meta-analysis can move beyond the rigid, one-size-fits-all approach to predict how an individual with specific characteristics and in a particular context might respond to specific active ingredients, which in turn may be more closely aligned with neuroscience (Craske *et al.*, 2023). Working with individual rather than aggregate data, with individual components rather than complex interventions, with a trans-diagnostic dimensional approach rather than the conventional categorical approach, with person-centered outcomes in addition to clinical outcomes, and with resource levels rather than standard income classifications to categorize contexts, global mental health research can be reimagined by moving mental health policy and practice towards person-centered care.
